# Supplementary material for: Ancient origin of fucosylated xyloglucan in charophycean green algae
Source: Commun Biol. 2021 Jun 17;4:754. doi: 10.1038/s42003-021-02277-w (PMC8211770; doi:10.1038/s42003-021-02277-w)
Supplement: Supplementary file 2 — Supplementary material [file 42003_2021_2277_MOESM2_ESM.pdf]

Supplementary tables and figures

Table S1 primers used for cloning of *CoGT47A1* and *2* genes

Gene	Name		Sequence
CoGT47A1	FW1		5’-GAGTGGGACGAGTGTGGGGTGTTGG-3’
	RV		5’-CCCCTCCACTACGCGATCCACCGCG-3’
	FW2		5’-CATAGCCCCAATCAGGGAGAATC-3’
	FW3n		5’-GGCTGCATCCCTGTTTTCTTCTG-3’
	FW4n		5’-CAGCCCGGACAAGATTCAAAAG-3’
	GSP1		5’-GATCATCATCTTCTCCACAGCGCGGC-3’
	NGSP1		5’-GTCCTTGGCATTGCTGTAGGAGATCG -3’
	FLFW		5’-ATGAGAAGGTGTACCAGAAACCCTC-3’
	FLRV		5’-CTAGGCAGTGCCCAGTGGCATTC-3’
CoGT47A2	FW1		5’-CCCGTACCCTGTCTTCTTCCATCCC-3’
	RV		5’-GATCATCATCTTCTCCACAGCGCGGC-3’
	FW2		5’-GCTGCTTGATGAGTGCGAGGGATGGG-3’
	GSP1		5’-GGCTCCTTGCATGGTGACTTCTCTC-3’
	NGSP1		5’-CTGGCAGTCCAAAACTCCACATTCC-3’
	FLFW		5’-ATGAAAAGGGCTGCCAAAAATCCTCTC-3’
	FLRV		5’-TTATGACGGCTGTTGTAGAGAAGCG-3’
Anchored oligo-dT primer			TTTTTTTTTTTTTTTTTTTTTTTV

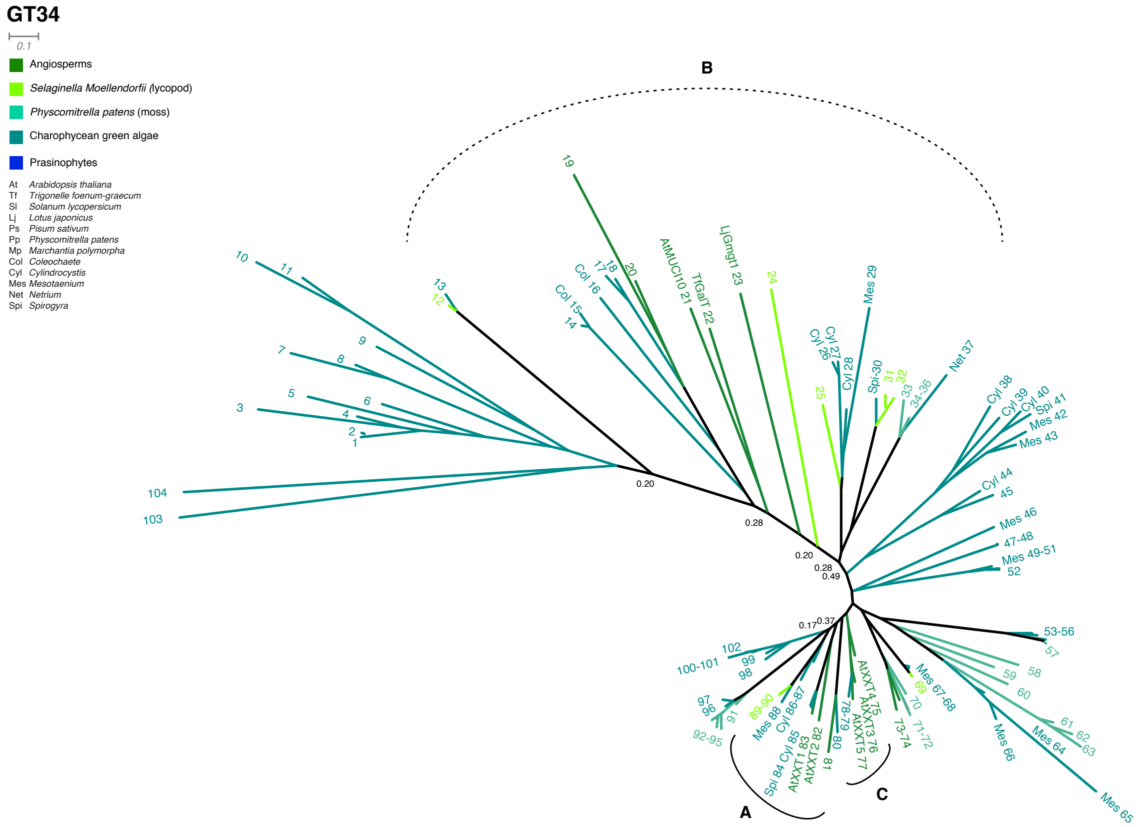

Figure S1 Phylogenetic analysis of CAZy-family GT34. Branches are color-coded according to taxonomic group. Bootstrap values below 0.7 are indicated along the backbone of the tree. Keys to the numbered sequences are found in supplemental File 1. The numbers are preceded by a three-letter genus prefix whenever a species belonging to the genus has been selected for MALDI-ToF analysis . Key to the genera is shown to the left in the figure. Sequences for which experimental evidence of function exist are indicated with their gene names: The A- and C-clades comprise *A. thaliana* XyG xylosyltransferases AtXXT1-5[1],[2]; the B-clade includes Galacto(gluco)mannan galactosyltranferases AtMUCl10 from *A. thaliana*[3], LjGmgt1 from *Lotus Japonicus*[4] and TfGalT from Trigonella foenum-graecum[5]. The poorly resolved B-clade is indicated with a dashed arc comprising sequences delimited by the previously annotated *Selaginella* B-clade sequences[6].

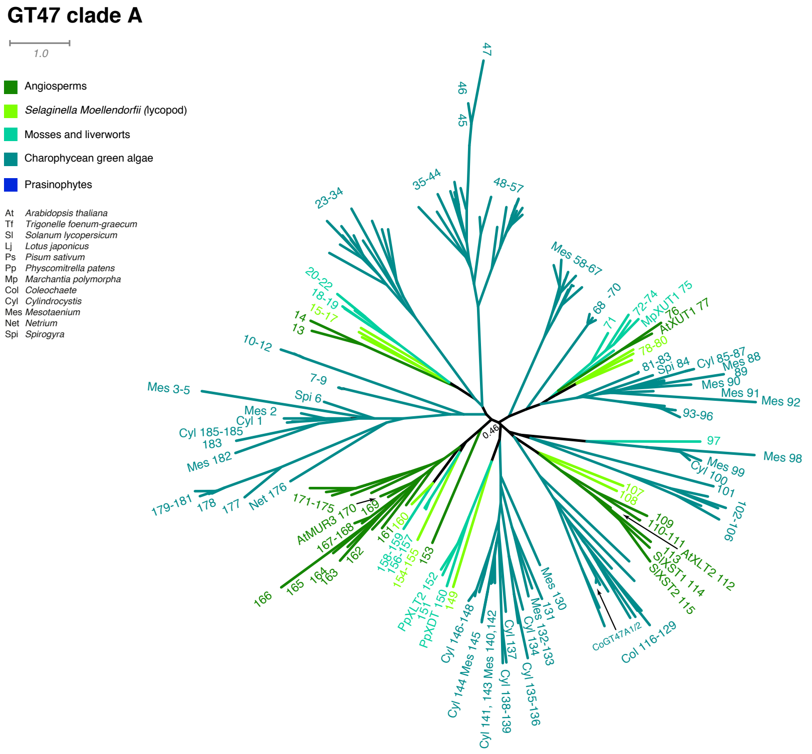

Figure S2 Phylogenetic analysis of the A-clade of CAZy-family GT47. Branches are color-coded according to taxonomic group. Sequences are numbered and the key to the tree is found in supplemental File 1. Numbers are preceded with three-letter prefixes, see key in the figure, for the species that are analyzed in detail by MALDI-ToF; and by gene names for those sequences where experimental evidence of function has been provided: Formation of the L-chain (and F-chain) in *A. thaliana* relies on galactosyltransferases, AtMUR3[7] and AtXLT2[8]. AtXUT1[9] and *Marchantia polymorpha* MpXUT1[10] encode XyG GalA-transferases thus forming the Y-chain and are prerequisite for P-chain formation. PpXDT from the moss *P. patens* transfers an Ara*p*-residue to the same position thus forming the D-chain[11], and tomato SlXST1 and SlXST2, encode Ara*f*-transferases forming the S-chain.

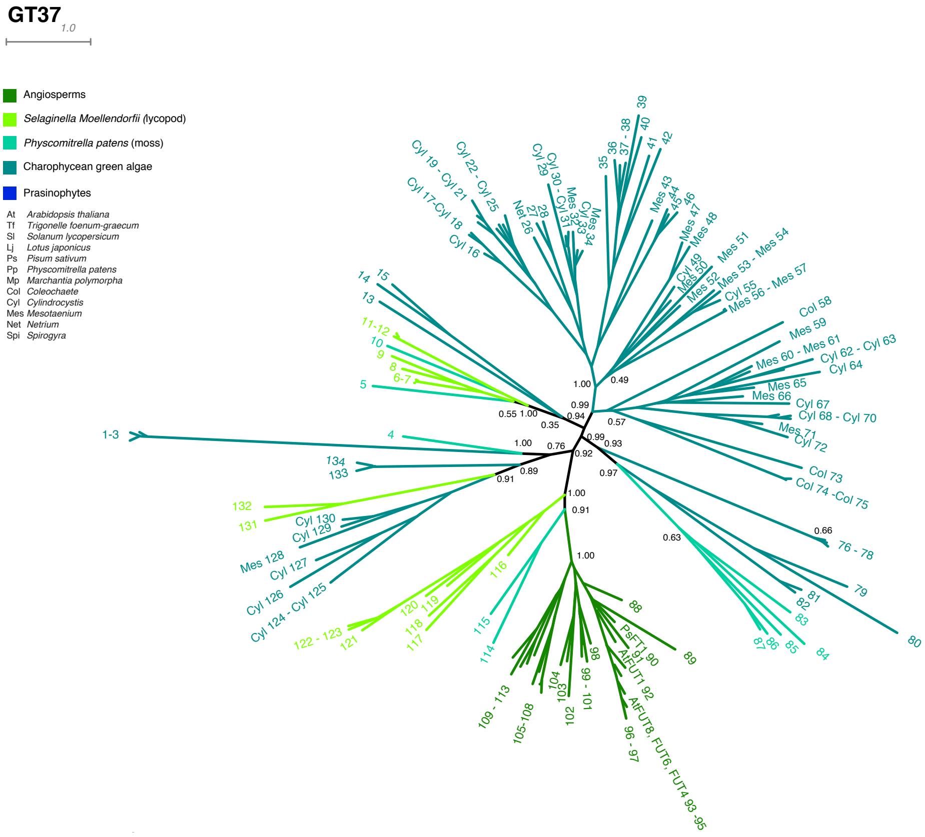

Figure S3 Phylogenetic analysis of CAZy-family GT37. Branches are color-coded according to taxonomic group. Bootstrap values below 0.7 are indicated along the backbone of the tree. Keys to the numbered sequences are found in supplemental File 1. The numbers are preceded by a three-letter genus prefix whenever a species belonging to the genus has been selected for MALDI-ToF analysis. Key to the genera is shown to the left in the figure. *Pisum sativum* XyG fucosyltransferase PsFT1[12]; *A. thaliana* XyG fucosyltransferase AtFUT1[13] and *A. thaliana* fucosyltransfeases AtFUTs using acceptor substrates other than XyG[14] of which AtFUT 4 and 6 have been characterized as arabinogalactan fucosyltransferases[15].

Antibody	LM25				LM15			
Extraction	CDTA		NaOH		CDTA		NaOH	
Plant species	-	E-XEGP	-	E-XEGP	-	E-XEGP	-	E-XEGP
Mesostigma viride	0	0	0	0	0	0	6	0
Chlorokybus atmophyticus	0	0	0	0	0	0	0	0
Entransia fimbriata	0	0	0	0	0	0	0	0
Klebsormidium flaccidum	0	0	0	0	0	0	0	0
Klebsormidium dissectum	0	0	0	0	0	0	0	0
Klebsormidium sp.	0	0	0	0	0	0	0	0
Chara corallina (apices)	0	0	0	0	36	36	37	36
Chara corallina	0	0	0	0	62	62	11	10
Chara corallina	0	0	0	0	62	62	17	12
Chara corallina	0	0	0	0	61	60	24	9
Chara corallina	0	0	0	0	37	37	37	35
Chara corallina	0	0	5	0	26	26	30	30
Chara corallina	0	0	0	0	41	43	38	38
Nitella sp.	0	0	73	9	25	22	76	11
Coleochaete nitellarum	0	0	0	0	29	28	38	19
Coleochaete nitellarum	0	0	0	0	32	33	37	35
Coleochaete scutata	0	0	0	0	0	0	0	0
Coleochaete orbicularis	0	0	0	0	0	0	0	0
Closterium acerosum	0	0	44	0	0	0	0	0
Closterium moniliferum	0	0	14	0	0	0	0	0
Closterium praelongum	0	0	19	0	0	0	25	12
Cosmarium sp.	0	0	0	0	33	33	37	36
Cosmarium turpini	0	0	0	0	34	31	14	18
Cosmarium turpini	0	0	0	0	34	28	22	18
Desmidium swartzii	0	0	0	0	0	0	0	0
Groenbladia undulata	0	0	0	0	0	0	6	0
Micrasterias furcata	0	0	0	0	0	0	0	0
Micrasterias radiata	0	0	0	0	0	0	0	0
Micrasterias sp.	0	0	0	0	0	0	0	0
Onychonema sp.	0	0	0	0	0	0	0	0
Pleurotaenium trabecula	0	0	0	0	0	0	8	7
Teilingia granulata	0	0	0	0	0	0	5	0
Tetmemorus brebissonii	0	0	0	0	0	0	0	0
Cylindrocystis brebissonii.	7	0	33	0	0	0	0	0
Mesotaenium caldariorum	0	0	48	0	40	39	59	25
Netrium sp.	21	0	85	0	19	0	100	0
Netrium sp.	0	0	38	0	25	23	50	0
Netrium digitus	0	0	56	0	7	0	19	9
Netrium digitus	0	0	78	0	0	0	5	0
Netrium interruptum	0	0	78	7	25	25	35	16
Netrium oblongum	5	0	36	0	9	0	0	0
Netrium sp.	0	0	10	0	0	0	9	0
Netrium interruptum	0	0	20	0	0	0	26	0
Netrium sp.	0	0	20	0	27	27	30	0
Penium spirostrialatum	0	0	0	0	0	0	0	0
Penium margaritaceum	0	0	0	0	0	0	0	0
Penium sp.	0	0	0	0	0	0	0	0
Mougeotia transeau	0	0	0	0	47	46	44	44
Spirogyra communis	9	0	33	25	80	78	80	63
Spirogyra sp.	16	7	50	12	41	57	27	23
Zygnema sp.	0	0	0	0	46	49	23	22

Figure S4 Heatmap of CoMPP of CGA species using sequential extraction and probing with XyG-specific antibodies LM15 and LM25

Antibody	LM25								LM15							
Extraction	CDTA				NaOH				CDTA				NaOH			
Enzyme treatment	-	E-XEGP	-	XGHA	-	E-XEGP	-	XGHA	-	E-XEGP	-	XGHA	-	E-XEGP	-	XGHA
Mesostigma viride	0	0	0	0	14	0	21	0	0	0	0	0	0	0	0	0
Chlorokybus atmophyticus	0	0	0	0	0	0	0	0	0	0	0	0	0	0	0	0
Entransia fimbriata	11	0	14	0	16	0	25	0	0	0	0	0	0	0	0	0
Klebsormidium flaccidum	0	0	0	0	6	0	8	0	0	0	0	0	0	0	0	0
Klebsormidium flaccidum	0	0	0	0	0	0	0	0	0	0	0	0	0	0	0	0
Klebsormidium dissectum	0	0	0	0	0	0	0	0	0	0	0	0	0	0	0	0
Chara corallina	0	0	0	0	10	0	12	0	24	21	25	27	17	18	19	20
Coleochaete nitellarum	19	11	20	8	92	68	100	63	23	22	26	28	24	20	30	24
Coleochaete orbicularis	0	0	0	0	31	5	36	0	0	0	0	0	12	0	13	0
Closterium acerosum	0	0	0	0	9	0	11	0	0	0	0	0	0	0	0	0
Cosmarium turpini	10	0	12	0	34	17	40	18	27	28	31	33	35	36	33	36
Micrasterias furcata	0	0	0	0	12	0	15	0	0	0	0	0	0	0	6	0
Pleurotaenium trabecula	0	0	0	0	0	0	0	0	0	0	0	0	0	0	0	0
Teilingia granulata	0	0	0	0	0	0	0	0	0	0	0	0	0	0	0	0
Tetmemorus brebissonii	9	0	11	0	14	5	9	5	0	0	0	0	0	0	0	0
Cylindrocystis brebissonii	10	0	9	0	53	0	59	7	0	0	0	0	8	0	8	0
Mesotaenium caldariorum	0	0	0	0	62	0	66	0	29	26	31	34	57	47	62	56
Netrium interruptum	5	0	0	0	34	0	36	0	23	22	26	25	43	7	45	8
Penium margaritaceum	5	0	6	0	18	5	27	6	0	0	0	0	0	0	0	0
Mougeotia transeau	0	0	0	0	0	0	0	0	47	49	54	58	51	53	63	69
Spirogyra communis	7	0	5	0	39	21	38	16	46	48	53	56	44	49	54	57

Figure S5 Heatmap of XyG antibody labelling of some CGA is xyloglucanase (E-XEGP) degradable but not more degradable when using XcXGHA from *Xanthomonas citri* pv. *mangiferaeindicae*, an endo-β-1,4-glucanase cleaving unsubstituted XyG at the -1 position between XX as well as XL sidechains. (Different pH was used for the two xyloglucanases).

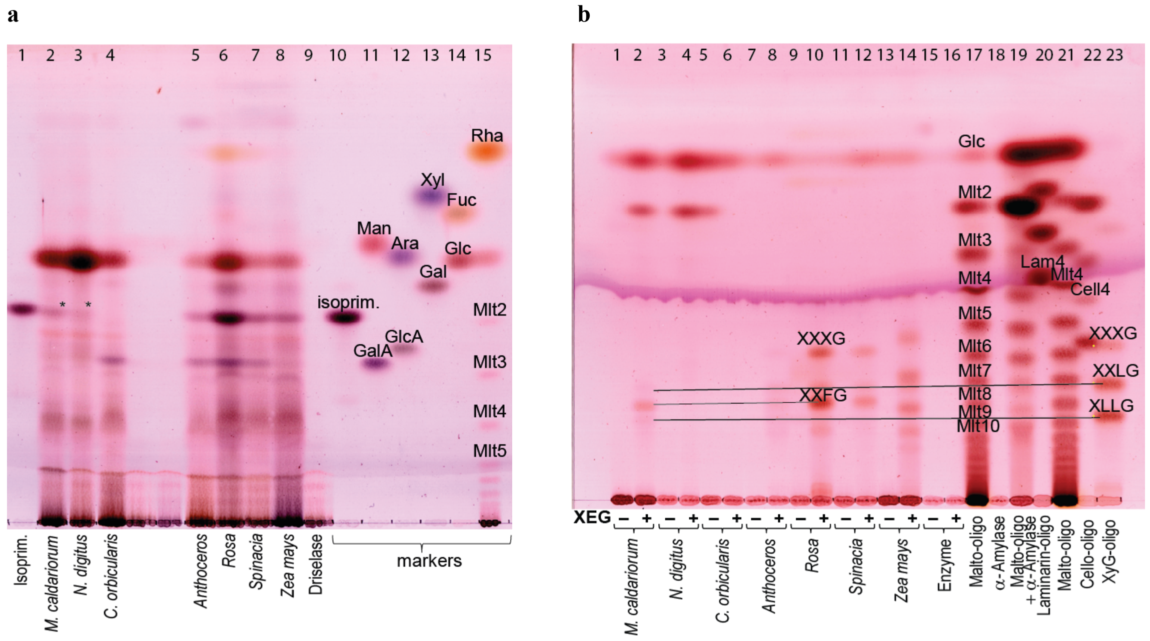

Figure S6 TLC analysis of enzymatically released isoprimeverose and XGOs from selected CGA.

a) Isoprimeverose is released by Driselase treatment of *M. caldariorum* and *N. digitus*. Cell wall material from three CGA and four land plants were treated with Driselase and released sugars were separated using TLC. Controls: 1) and 10) isoprimeverose; 9) Driselase autolysis. Plant samples treated with Driselase 2) *M. caldariorum*, 3) *N. digitus*, 4) *C. orbicularis,* 5) the hornwort *Anthoceros caucasicus*, 6) *Rosa* sp., 7) *Spinacia oleracea*, 8) *Zea mays*. Control 9) Driselase autolysis products. Markers used were 1) and 10), isoprimeverose; 11) mannose (Man) and galacturonic acid (GalA), 12) arabinose (Ara) and glucuronic acid (GlcA), 13) galactose (Gal) and xylose (Xyl), 14) glucose (Glc) and fucose (Fuc), 15) Rhamnose (Rha), maltose-oligosaccharides and glucose (Mlt, Glc). * indicate Driselase-released isoprimeverose in *M. caldariorum* and *N. digitus*. b) Xyloglucanase digest of CGA species results in release of putative fucosylated XGOs in *M. caldariorum*. Cell wall material from three CGA and four land plants was treated with xyloglucanase followed by α-amylase, and released XGOs were separated by TLC. Plant samples treated with (+) or without (–) XEG (xyloglucanase) include the CGA 1-2) *M. caldariorum*, 3-4) *N. digitus*, 5-6) *C. orbicularis*, the hornwort 7-8) *Anthoceros* as well as the flowering plants 9-10) *Rosa*, 11-12) *Spinacia*, 13-14) *Zea mays*. Control 15-16) XEG only. Markers used were 17 and 21) glucose and maltose-oligosaccharides (Glc, Mlt), 18) α-amylase alone, 19) α-amylase-treated glucose and maltose-oligosaccharides, 20) laminarin-oligosaccharides incl. glucose, 22) cellulose-oligosaccharides (cell2-5), and 23) XyG-oligosaccharides (XLLG, XXLG/XLXG, and XXXG). Bands corresponding to XLLG, XXFG and XXLG/XLXG are released in *M. caldariorum* after XEG treatment (lane 2).

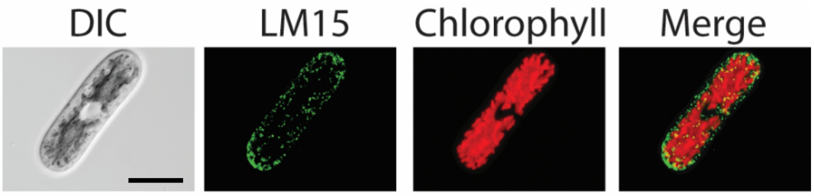

Figure S7 XyG antibody labelling of fully developed *Cylindrocystis brebissonii* cells. XyG labelling with LM15 is visible in a spotted pattern in the cell wall. DIC: light image, LM15 (TRITC) (green), chlorophyll autofluorescence (red). Merge: LM15 and chlorophyll autofluorescence. Scalebar: 20 µm as indicated in the DIC image.

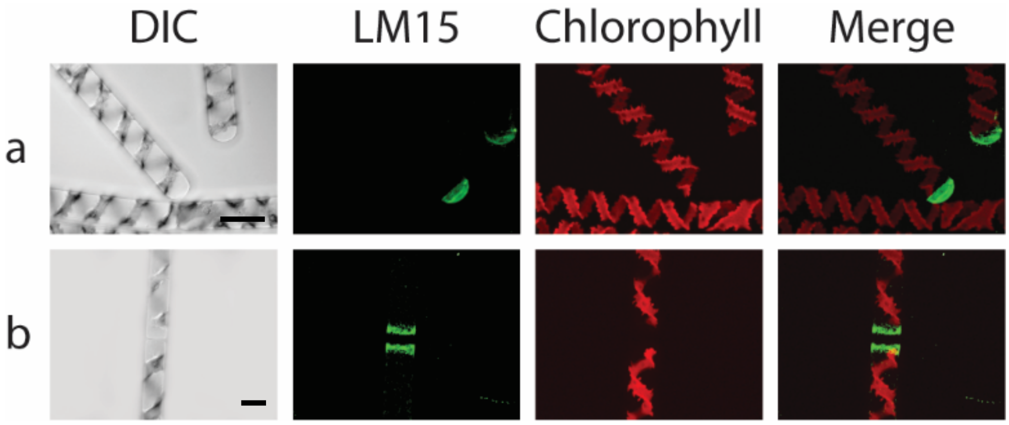

Figure S8. XyG immunolabeling in *Spirogyra communis*. a) LM15 signal is restricted to the cell walls of tip of growing filaments and to b) the cell walls of cross-wall zones between cells of the filaments, likely involved in cell expansion. DIC: light image, LM15 (TRITC)(green), chlorophyll autofluorescence (red). Merge: LM15 and chlorophyll autofluorescence. Scalebar: 20 µm as indicated in the DIC image in each row.

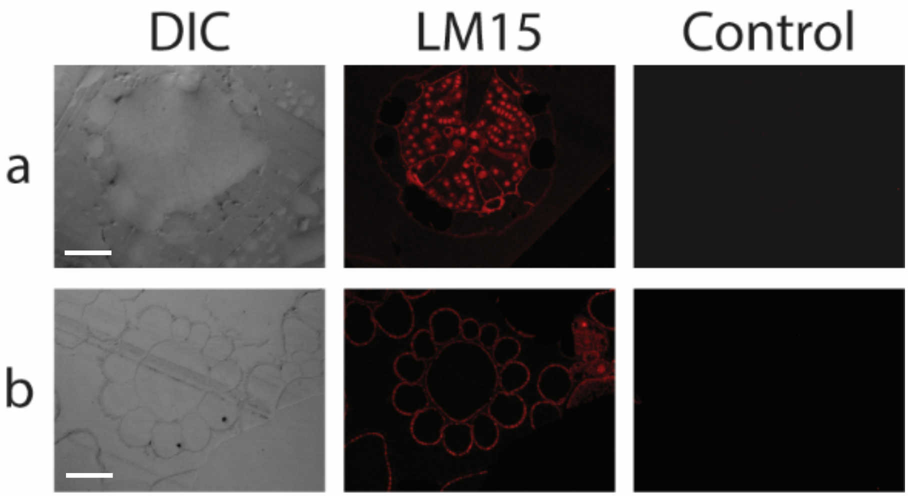

Figure S9 XyG immunolabeling of *Chara corallina*. LM15 labels to the cell wall of a) developing antheridium and b) young apical zones in *Chara corallina*. These tissues are undergoing elaborate cell divisions and elongation. These images were made on embedded tissues devoid of chlorophyll. DIC: light image, LM15 (TRITC) (red), control: chlorophyll autofluorescence. Scalebar: 50 µm as indicated in the DIC image in each row.

	M1				M39			
	-	E-XEGP	-	Mfuc5	-	E-XEGP	-	Mfuc5
Netrium digitus	0	0	0	0	0	0	0	0
Cylindrocystis brebissonii	0	0	0	0	0	0	0	0
Coleochaete orbicularis	0	0	0	0	0	0	0	0
Mesotaenium caldariorum	54	0	59	0	80	0	100	6

Figure S10 CoMPP heatmap analysis of fucosylated XyG in NaOH extracts. Fucosyl-specific antibodies CCRC-M1 and M39 antibodies were used. Labeling removed by fucosidase treatment in *M. caldariorum* Different pH was used for the two enzymes

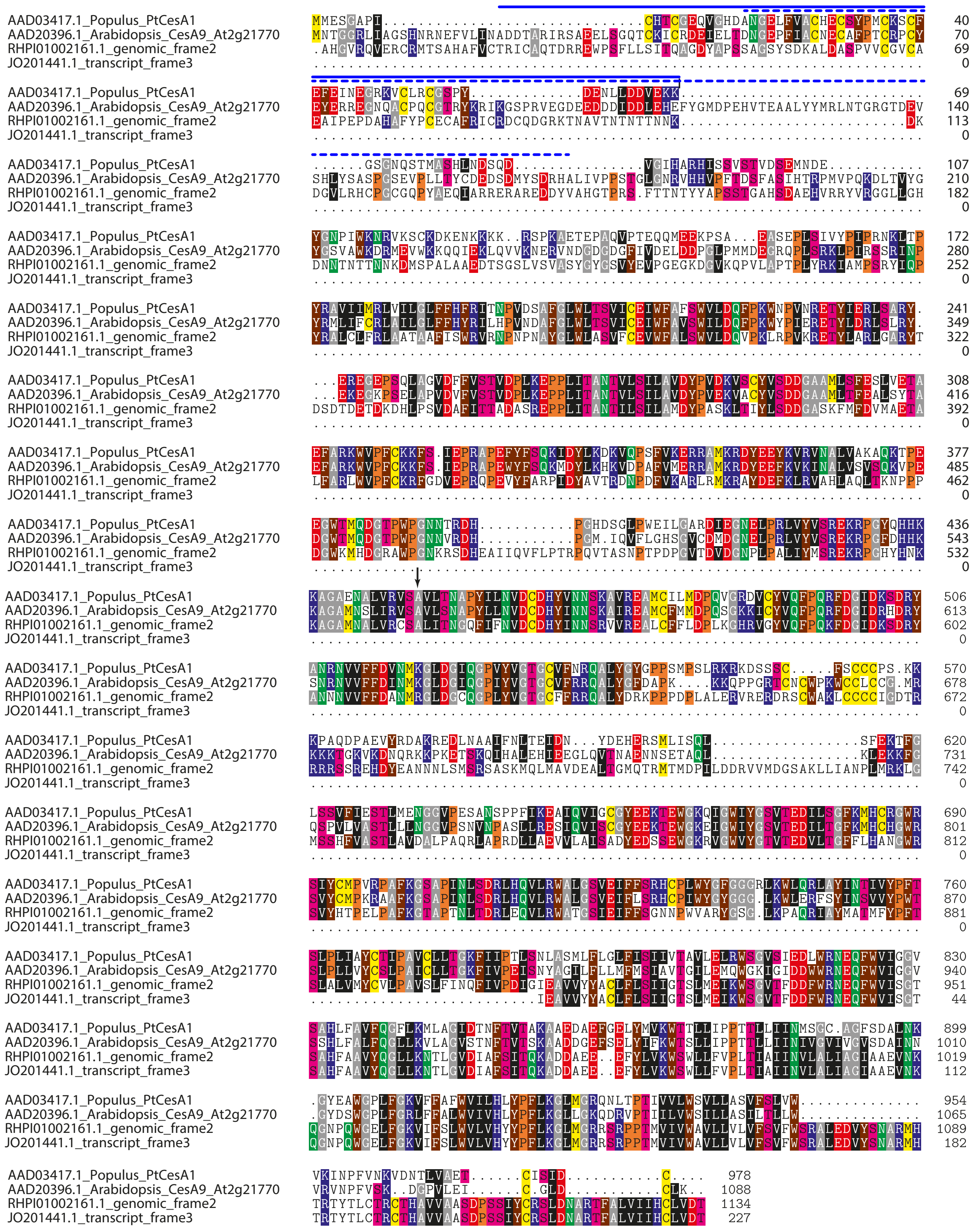

Figure S11 Alignment of a *Populus tremuloides* PtCesA1, *A. thaliana* AtCesA9 a translated genomic *Chlorokybus atmophyticus* sequence identified in RHPI01002161.1 and a translated *Chlorokybus* transcript [16]. The Zinc binding domain implicated in rosette formation as predicted by InterProScan [17] is indicated as a solid blue line for AtCesA9 and a dashed line for the *Chlorokybus* sequence. The arrow points to an alanine residue that, when substituted with valine in the *A. thaliana* rsw1 mutant, led to less stable rosettes [18].

References

1. Zabotina, O. A. *et al.* Mutations in multiple *XXT* genes of arabidopsis reveal the complexity of xyloglucan biosynthesis. *Plant Physiol.* **159**, 1367–1384 (2012).

2. Vuttipongchaikij, S. *et al. Arabidopsis* GT34 family contains five xyloglucan α-1,6-xylosyltransferases. *New Phytol.* **195**, 585-595 (2012).

3. Voiniciuc, C. *et al.* MUCILAGE-RELATED10 produces galactoglucomannan that maintains pectin and cellulose architecture in arabidopsis seed mucilage. *Plant Physiol.* **169**, 403–420, (2015).

4. Edwards, M. E. *et al.* The seeds of *Lotus japonicus* lines transformed with sense, antisense, and sense/antisense galactomannan galactosyltransferase constructs have structurally altered galactomannans in their endosperm cell walls. *Plant Physiol.* **134**, 1153–1162 (2004).

5. Edwards, M. E. *et al.* Molecular characterisation of a membrane-bound galactosyltransferase of plant cell wall matrix polysaccharide biosynthesis. *Plant J.* **19**, 691-697 (1999).

6. Harholt, J. *et al.* The glycosyltransferase repertoire of the spikemoss *Selaginella moellendorffii* and a comparative study of its cell wall. *PLoS One* **7**, e35846 (2012).

7. Madson, M. *et al.* The *MUR3* gene of *Arabidopsis* encodes a xyloglucan galactosyltransferase that is evolutionarily related to animal exostosins. *Plant Cell* **15**, 1662-1670 (2003).

8. Jensen, J. K., Schultink, A., Keegstra, K., Wilkerson, C. G. & Pauly, M. RNA-seq analysis of developing nasturtium seeds (*Tropaeolum majus*): Identification and characterization of an additional galactosyltransferase involved in xyloglucan biosynthesis. *Mol. Plant* **5**, 984-992 (2012).

9. Peña, M. J., Kong, Y., York, W. S. & O'Neill, M. A. A galacturonic acid-containing xyloglucan is involved in *Arabidopsis* root hair tip growthw. *Plant Cell* **24**, 4511–4524 (2012).

10. Honkanen, S. *et al.* The Mechanism Forming the Cell Surface of Tip-Growing Rooting Cells Is Conserved among Land Plants. *Curr. Biol.* **26**, 3238–3244 (2016).

11. Zhu, L., Dama, M. & Pauly, M. Identification of an arabinopyranosyltransferase from *Physcomitrella patens* involved in the synthesis of the hemicellulose xyloglucan. *Plant Direct* 1-9 (2018).

12. Perrin, R. M. *et al.* Xyloglucan fucosyltransferase, an enzyme involved in plant cell wall biosynthesis. *Science* **284**, 1976-1979 (1999).

13. Vanzin, G. F. *et al.* The *mur2* mutant of *Arabidopsis thaliana* lacks fucosylated xyloglucan because of a lesion in fucosyltransferase AtFUT1. *Proc. Natl. Acad. Sci. U. S. A.* **99**, 3340-3345 (2002).

14. Sarria, R. *et al.* Characterization of a family of Arabidopsis genes related to xyloglucan fucosyltransferase1. *Plant Physiol.* **127**, 1595–1606 (2001).

15. Tryfona, T. *et al.* Characterisation of FUT4 and FUT6 α-(1→2)-fucosyltransferases reveals that absence of root arabinogalactan fucosylation increases arabidopsis root growth salt sensitivity. *PLoS One* **9**, e93291 (2014).

16. Timme, R. E., Bachvaroff, T. R. & Delwiche, C. F. Broad phylogenomic sampling and the sister lineage of land plants. *PLoS One* **7**, e29696 (2012).

17. Jones, P. *et al.* InterProScan 5: Genome-scale protein function classification. *Bioinformatics* **30**, 1236-1240 (2014).

18. Arioli, T. *et al.* Molecular analysis of cellulose biosynthesis in Arabidopsis. *Science* **279**, 717-720 (1998).
