# Supplementary material for: Ancient origin of fucosylated xyloglucan in charophycean green algae
Source: Commun Biol. 2021 Jun 17;4:754. doi: 10.1038/s42003-021-02277-w (PMC8211770; doi:10.1038/s42003-021-02277-w)
Supplement: Supplementary file 3 — Description of Additional Supplementary Files [file 42003_2021_2277_MOESM3_ESM.pdf]

Description of Additional Supplementary Files

File name: Supplementary Data 1

Description: Key to phylogenetic trees and raw data underlying CoMPP analyses.
